# A pilot assessment of the career and job satisfaction of the pharmaceutical workforce in Lebanon

**DOI:** 10.1186/s40545-022-00498-w

**Published:** 2022-11-28

**Authors:** Elsa Nassar, Sibelle Kassouf, Aline Hajj, Hala Sacre, Marwan Akel, Rony M. Zeenny, Chadia Haddad, Pascale Salameh

**Affiliations:** 1 Faculty of Pharmacy, Lebanese University, Hadat, Lebanon; 2 INSPECT-LB (Institut National de Santé Publique, d’Épidémiologie Clinique et de Toxicologie-Liban), Beirut, Lebanon; 3 Laboratoire de Pharmacologie, Pharmacie Clinique et Contrôle de Qualité des Médicament, Faculty of Pharmacy, Saint Joseph University of Beirut, Beirut, Lebanon; 4 Faculté de Pharmacie, Université Laval, Québec, Canada; 5 Oncology Division, CHU de Québec- Université Laval Research Center, Québec city, Québec, Canada; 6 School of Pharmacy, Lebanese International University, Beirut, Lebanon; 7 Department of Pharmacy, American University of Beirut Medical Center, Beirut, Lebanon; 8 School of Medicine, Lebanese American University, Beirut, Lebanon; 9 Research Department, Psychiatric Hospital of the Cross, P.O. Box 60096, Jal Eddib, Lebanon; 10 School of Health Sciences, Modern University for Business and Science, Beirut, Lebanon; 11 Department of Primary Care and Population Health, University of Nicosia Medical School, 2417 Nicosia, Cyprus

**Keywords:** Pharmacy workforce, Pharmacist, Healthcare, Job satisfaction, Pharmacy, Crisis

## Abstract

**Background:**

Lebanon has been recognized as a center for high-quality healthcare services in the Middle East because of its prestigious facilities certified by international bodies, competent health workers, and credible pharmaceutical sector. This study assessed the professional situation of the Lebanese pharmaceutical workforce upon graduation and entry to the job market. It also evaluated the satisfaction of pharmacists with the financial, physical, and psychological aspects of their job and the effect of the current socioeconomic crisis on their profession.

**Methods:**

A cross-sectional study was performed between January and May 2021 among 114 Lebanese pharmacists from all pharmaceutical sectors across Lebanon. A self-administered questionnaire was elaborated to assess the pharmaceutical workforce in Lebanon. The online questionnaire was built using Google Forms and disseminated via emails and social platforms.

**Results:**

Fresh graduates seemed more oriented toward community pharmacies, and 78.1% of the participants worked at least once in their lifetime in a community pharmacy. Pharmacists from the public university worked predominantly in the community pharmacy sector, while those who graduated from private universities worked mainly as medical representatives. Hospital pharmacy comprised significantly more PharmD graduates than other sectors; medical representatives had mainly a BS pharmacy, while those working as industrial pharmacists had a Master’s degree. A low percentage (31.6%) of participants found it easy to get hold of a job across different pharmaceutical sectors while 64.0% considered the internships experience insufficient to get employed. Nearly half (48.2%) of the respondents were poorly satisfied with their job, and 54.0% of community pharmacists reported not working in their preferred field compared to 39.1% of pharmacists from other sectors. Also, 65.79% felt that the economic crisis and the consequent medication shortages affected their credibility and how society perceived them. About half (45.61%) of the participants reported that their employment status was not affected by the crisis; the rest got a second job to cover their expenses (15.79%) or changed jobs (14.91%).

**Conclusion:**

Our study findings revealed that most Lebanese fresh graduates worked as community pharmacists, which does not correspond to their preference, the available job market, and the modern pharmacy wingspan. Most pharmacists were also financially, physically, and emotionally dissatisfied due to the Lebanese economic crisis that added heavy workloads and responsibilities without any financial raise.

## Introduction

The World Health Organization (WHO) defines the health workforce as a critical building block for robust health systems and provides an understanding of the workforce quality, accessibility, acceptability, and availability requirements [1]. The International Pharmaceutical Federation (FIP) defines the pharmaceutical workforce as the whole of the pharmacy-related workforce (e.g., registered pharmacist practitioners, pharmaceutical scientists, pharmacy technicians, and other pharmacy support workforce categories and pre-service students/trainees) working in different settings (e.g., community, hospital, research and development, industry, military, regulatory, academia, and any other public or private sectors) with a diversity of scope of practice [2]. Pharmaceutical Workforce Development Goals as set by the FIP are monitoring, progressing, and enabling [3].

Pharmacists play a significant part in the provision of healthcare services. However, all sectors have reported a lack of pharmacists in the workforce [4]. In addition to the rising need for healthcare workers globally, the pharmacy profession is changing quickly with a greater emphasis on patient-centered care, clinical decision-making on medicine use, and interprofessional collaboration [5]. The number of pharmacists working and the availability of pharmacists worldwide varies considerably by country [5]. Globally, countries with fewer pharmacists per capita are probably less likely to have access to medications and less availability of pharmaceutical services and advice [5].

The 2015 and 2018 FIP reports on the pharmaceutical workforce in the Eastern Mediterranean region showed an increase in market capacity compared to other parts of the world [6]. Several studies showed an increasing number of pharmacy schools in some countries of this region without strategic planning for the socioeconomic needs of the health system at the national level [7], highlighting a disparity between the education, regulation, and practice sectors.

Lebanon has been recognized as a center for high-quality healthcare services in the Middle East because of its prestigious facilities certified by international bodies, competent health workers, and credible pharmaceutical sector [8]. The Lebanese pharmaceutical market institutions encompasse 230 importers, 12 local manufacturers, and 32 scientific offices [9]. In addition, hospital pharmacy has a limited number of positions, since hospitals have to hire at least one hospital pharmacist as required by the law (*n* = 130 overall), while big university hospitals who need more than one hospital pharmacist are not numerous (9 in total). The newest records of the Order of Pharmacists of Lebanon (OPL) reveal a current total number of 6951 pharmacists (out of 10,394 registered) practicing in different areas of Lebanon and 3398 licensed pharmacies serving a population of seven million individuals approximately [10]: each community pharmacy in Lebanon is the property of one or more pharmacists, while chain pharmacies are legally forbidden. A community pharmacist may also manage or be employed in a community pharmacy, without being the owner of it. Given the abovementioned figures, the Lebanese pharmaceutical sector offers the graduates various career paths as they can choose to be community/hospital pharmacists, clinical pharmacists, or pharmacists employed in different departments of pharmaceutical companies (e.g., sales, marketing, policy, and research and development, among others), or pharmacists employed in academic or governmental institutions. Community pharmacies (60 to 76%) are the most common employer of pharmacists in Lebanon across all age categories, followed by pharmaceutical companies (12 to 28%) [11]. Pharmacists cannot work as full timers in more than one institution; only teaching is allowed as per the Lebanese laws.

In Lebanon, the number of fresh graduates is continually increasing without any adaptation to the market needs, whether from educational institutions or regulatory authorities, and has doubled in the past 10 years. However, these figures did not consider employed pharmacists not registered with the OPL and pharmacy students working part-time jobs in the pharmaceutical sector. Accordingly, the already high enough influx of pharmacists on the market is underestimated. A study from 2017 found that the ratio of pharmacists per 10,000 people in Lebanon was 17.52, with a potential of 41.17 pharmacists per 10,000 people by 2050, compared to six pharmacists per 10,000 inhabitants in a study on 80 countries, three times lower than the Lebanese mean ratio of pharmacists [11]. The oversupply of graduates is raising competition among pharmacists and decreasing the demand (and hence salaries for hired pharmacists), thus resulting in a lowered quality of services provided to patients.

Besides these numbers underlining a highly probable unemployment problem, employed pharmacists reported being dissatisfied at the financial, physical, and psychological levels [12]. Most community pharmacists were satisfied with the social benefits of their work, their image in society, and their moral and intellectual contribution to patient care but were dissatisfied with their financial situation. While pharmacists working in pharmaceutical companies were financially satisfied, they were not happy with their image in society mainly because medical promotion is practiced by medical and non-medical individuals, leading to an underestimation of their scientific background. Also, pharmacists working in hospitals were primarily dispensing medications rather than practicing patient-centered and clinical roles due to a lack of job opportunities, support from some physicians, and appropriate health policies [12–14]. Furthermore, it is noteworthy that Lebanon is going through one of the most severe socioeconomic crises of its history (loss of more than 90% of the Lebanese Pound value in 2 years), which is affecting the entire health system, particularly the pharmaceutical sector. Unquantified damages include increased unemployment rates and further worsening of pharmacists’ socioeconomic levels.

It is thus crucial to plan and develop the pharmaceutical workforce to maintain access to high-quality health services, improve health outcomes, strengthen the healthcare system [15], and enhance preparedness for emergencies. Efforts are mainly required in low- and middle-income countries that lack good socioeconomic management of the workforce capacity to fulfill the health system needs and prevent personnel shortages in some areas, unemployment, and financial hardships [16].

Therefore, this study assessed the professional situation of the Lebanese pharmaceutical workforce upon graduation and entry to the job market. It also evaluated the satisfaction of pharmacists with the financial, physical, and psychological aspects of their job and the effect of the current socioeconomic crisis on their profession.

## Methods

### Study design and sample

A cross-sectional study was performed between January and May 2021 among Lebanese pharmacists from all pharmaceutical sectors across Lebanon. Registered and non-registered pharmacists with any degree who graduated from less than 10 years, practicing in any pharmaceutical setting across Lebanon, or not working were eligible to participate. Non-working pharmacists answered additional specific questions. Working pharmacists not registered with the OPL were recruited by sharing the survey on WhatsApp and professional Facebook groups, after a personal contact; these were mainly fresh graduates who did not complete their OPL registration yet. The sample did not include non-Lebanese pharmacists and undergraduates.

### Sample size calculation

Given that the effect of the current crisis effect on the pharmaceutical sector has not been quantified, this study is considered a pilot study. Assuming that more than 90% of pharmacists were affected by the current crisis, taking an alpha value of 5%, a beta value of 20%, an acceptable margin of error of 6%, and a design effect of 1 (simple random sampling), a minimum of 95 pharmacists was calculated using Epi-Info 7. Thus, an overall sample of 120 participants was targeted to account for possible missing data.

The sample was selected from the list of registered pharmacists provided by the OPL (total *n* = 6951), with 100 names being targeted in systematic random sampling: 1/70 pharmacists; if they did not fulfil the inclusion criterion of graduation from less than 10 years, they were replaced by the next name before or after it. After a personal contact with 100 eligible pharmacists, 95% responded. Moreover, 20 non registered pharmacists were targeted and contacted, and 19 (95%) responded. The obtained total sample was 114 pharmacists. No incomplete response was found because questions were mandatory.

### Questionnaire

A self-administered questionnaire was elaborated to assess the pharmaceutical workforce in Lebanon. The online questionnaire was built using Google Forms and shared via e-mails and social platforms, after a personal initial contact with the eligible pharmacist.

The questionnaire was divided into two parts and adapted from previous similar studies [14, 17]. The first part consisted of social and demographic characteristics of participants, such as gender, age, employment and residence region, graduation year, and university. It also collected information on the professional history and current professional situation. Structured dichotomous questions were mainly used in this section.

The second section focused on specific difficulties that might affect the professional situations of pharmacists from different sectors. This part was designed to assess the pharmacists’ opinion of employment opportunities, career perception, their satisfaction with the financial, physical, and psychological aspects of their job, and the responsibility of OPL in improving laws and providing job opportunities. Some questions were rated on a 5-point Likert scale, with values ranging from 1 (not true/strongly disagree) to 5 (very true/strongly agree); some others were dichotomous and were coded 1 and 0 for positive and negative answers, respectively. Higher scores indicated higher agreement with the suggested statements. Open questions were also included to allow respondents to describe their needs and concerns, if any. Finally, pharmacists currently unemployed answered specific questions about the reason for their unemployment and their current situations.

The final questionnaire was pre-tested on 10 pharmacists; no significant changes were made to the questions, as they were judged to be clear. The results of the pre-test were not included in the final database. The Cronbach alpha related to the satisfaction of pharmacists with the financial, physical and psychological aspects of their job was 0.78, which was considered appropriate.

### Statistical analysis

Data were analyzed on IBM SPSS version 23.0. A descriptive analysis was conducted using proportion, mean, and standard deviation. Chi-squared test was applied for bivariate analysis of categorical data, and a mean count was performed for continuous variables (age). Variables taken into account included gender, marital status, university, graduation year, degree (s), and working sector. Different questions were analyzed according to practice fields. A *p*-value ≤ 0.05 was considered statistically significant. Unplausible responses were coded as missing.

## Results

### Socio-demographic characteristics of the participants

Our sample included more females than males (60.5% vs. 39.5%), and the respondents were mainly single (74.6%). The mean age was 28.04 ± 5.17 years. These characteristics were not significantly different across the practice fields. Of the total sample, eight pharmacists declared being unemployed (7.02%).

Fresh graduates seemed more oriented toward community pharmacy, and 78.1% of the participants worked in this sector at least once in their lifetime; currently, 40.3% work in community pharmacy. This practice field was not limited to participants with a BS pharmacy; it also included pharmacists holding various postgraduate degrees. Pharmacists from the public university worked predominantly in the community pharmacy sector (42%), while those who graduated from one of the five private universities in Lebanon worked mainly as medical representatives, the Lebanese American University in particular (43.8%; *p* < 0.001). High percentages of newly graduates worked outside the health sector (52.4% if graduated 1–2 years ago, 38.1% if 3–4 years, versus 9.5% if 5–6 years and none if more than 7 years; *p* = 0.058).

Hospital pharmacy comprised significantly more PharmD graduates than other sectors; medical representatives had mainly a BS pharmacy, while those working as industrial pharmacists had a Master’s degree (*p* < 0.001) (Table 1).

**Table 1:** Social and demographic characteristics of participants (*n* = 114)

Characteristics of pharmacists	Total pharmacists	Community pharmacists	Hospital pharmacists^a^	Medical representatives	Industrial pharmacists^b^	Other sectors^c^	Not in healthcare^d^	*p*-value
*N* (%)	114 (100.0%)	50 (40.3%)	11 (8.9%)	16 (12.9%)	14 (11.3%)	12 (9.7%)	21 (16.9%)	
Gender								.26^e^
M (%)	45 (39.5%)	24 (48.0%)	3 (27.3%)	8 (50.0%)	5 (35.7%)	2 (16.7%)	7 (33.3%)	
F (%)	69 (60.5%)	26 (52.0%)	8 (72.7%)	8 (50.0%)	9 (64.3%)	10 (83.3%)	14 (66.7%)	
Age								
Mean (SD)	28.04 (5.17)	29.54 (6.67)	28.64 (4.59)	27.50 (3.54)	26.00 (2.53)	28.00 (3.33)	25.52 (1.81)	0.18^e^
Median	26.00	26.00	26.00	26.00	26.00	27.50	25.00	
Marital status								.24^e^
Single (%)	85 (74.6%)	35 (70.0%)	10 (90.9%)	10 (62.5%)	10 (71.4%)	11 (91.7%)	18 (85.7%)	
Married (%)	29 (25.4%)	15 (30.0%)	1 (9.1%)	6 (37.5%)	4 (28.6%)	1 (8.3%)	3 (14.3%)	
University								**< 0.001**^e*^
Lebanese University	45 (39.5%)	21 (42.0%)	2 (18.2%)	2 (12.5%)	4 (28.6%)	4 (33.3%)	17 (81.0%)	
Saint-Joseph University	20 (17.5%)	9 (18.0%)	5 (45.5%)	2 (12.5%)	4 (28.6%)	0 (0.0%)	0 (0.0%)	
Lebanese American University	9 (7.9%)	2 (4.0%)	3 (27.3%)	2 (12.5%)	1 (7.1%)	1 (8.3%)	0 (0.0%)	
Lebanese International University	23 (20.2%)	8 (16.0%)	0 (0.0%)	7 (43.8%)	5 (35.7%)	2 (16.7%)	3 (14.3%)	
Beirut Arab University	13 (11.4%)	6 (12.0%)	1 (9.1%)	3 (18.8%)	0 (0.0%)	4 (33.3%)	1 (4.8%)	
Abroad	4 (3.5%)	4 (8.0%)	0 (0.0%)	0 (0.0%)	0 (0.0%)	1 (8.3%)	0 (0.0%)	
Graduated since								0.058^e^
1–2 years	46 (40.3%)	19 (38.0%)	4 (36.4%)	6 (37.5%)	6 (42.9%)	3 (25.0%)	11 (52.4%)	
3–4 years	30 (26.3%)	11 (22.0%)	4 (36.4%)	4 (25.0%)	5 (35.7%)	3 (25.0%)	8 (38.1%)	
5–6 years	16 (14.1%)	5 (10.0%)	0 (0.0%)	3 (18.8%)	2 (14.3%)	5 (41.7%)	2 (9.5%)	
> 7 years	22 (19.3%)	15 (30.0%)	3 (27.3%)	3 (18.8%)	1 (7.1%)	1 (8.3%)	0 (0.0%)	
Highest educational level								
Bachelor	33 (28.9%)	19 (38.0%)	1 (9.1%)	9 (56.3%)	2 (14.3%)	1 (8.3%)	4 (19.0%)	**< 0.001**^e*^
Pharm D	22 (19.4%)	8 (16.0%)	7 (63.6%)	1 (5.9%)	0 (0.0%)	4 (33.3%)	4 (1.0%)	
Pharm D + Masters	24 (21.0%)	11 (22.0%)	3 (27.3%)	2 (12.5%)	4 (28.6%)	0 (0.0%)	5 (23.8%)	
Masters	35 (30.7%)	12 (24.0%)	0 (0.0%)	4 (25.0%)	8 (57.1%)	7 (58.3%)	8 (38.1%)	
Registered in OPL	95 (83.3%)	43 (86.0%)	10 (90.9%)	13 (81.3%)	11 (78.6%)	10 (83.3%)	16 (76.2%)	0.9^e^

^a^Hospital pharmacists include clinical pharmacists and employees of the hospital's pharmacy;

^b^Industrial pharmacists include pharmacists working in production, quality assurance and control, research and development, and scientific offices;

^c^Other sectors include academic pharmacists, governmental positions, NGOs positions;

^d^Not in healthcare are either unemployed or are working in non-healthcare institutions;

^e^Chi-square test

^*^Numbers in bold are statistically significant results

**Fig. 1:**
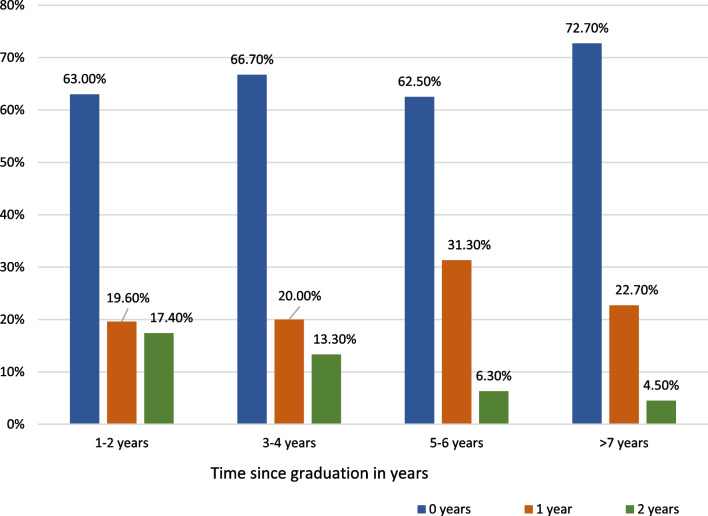
Needed time to find a full time job according to graduation year (*p* = 0.785)

Also, 54.0% of community pharmacists reported not currently working in their preferred field compared to 39.1% of pharmacists from other sectors. Additionally, the shorter the time since graduation, the longer the time needed to find a job. However, these results were not statistically significant (*p* = 0.785 > 0.05) (Fig. 1).

### Professional orientation and employment opportunities

A low percentage (31.6%) of participants found it easy to get hold of a job across different pharmaceutical sectors, while 64% considered the internships experience insufficient to get employed (Table 2). Moreover, 76.3% of participants believed that employment opportunities are being snatched by non-pharmacists who fill pharmacist positions for lower wages.

**Table 2:** Affirmative opinions regarding professional orientation and employment opportunities

Variable	Total pharmacists	Community pharmacists	Hospital pharmacists^a^	Medical representatives	Industrial pharmacists^b^	Other sectors^c^	Not in healthcare^d^	*p*-value
*N* (%)	114 (100.0%)	50 (40.3%)	11 (8.9%)	16 (12.9%)	14 (11.3%)	12 (9.7%)	21 (16.9%)	
Is it easy to find a job as a pharmacist?	36 (31.6%)	13 (26.0%)	6 (54.5%)	6 (37.5%)	5 (35.7%)	5 (41.7%)	4 (19.0%)	0.301^e^
Are internships enough to find a job	41 (36.0%)	14 (28.0%)	6 (54.5%)	6 (37.5%)	2 (14.3%)	2 (16.7%)	13 (61.9%)	0.008^e^
Is it possible to find job opportunities in all sectors?	47 (41.2%)	17 (34.0%)	5 (45.5%)	7 (43.8%)	7 (50.0%)	5 (41.7%)	9 (42.9%)	0.860^e^
Is pharmacists’ employment affected by non-pharmacists occupying their jobs?	87 (76.3%)	38 (76.0%)	7 (63.6%)	13 (81.3%)	12 (85.7%)	4 (33.3%)	21 (100.0%)	0.001^e^
Is there an orientation from universities for fresh graduates?	29 (25.4%)	10 (20.0%)	5 (45.5%)	7 (43.8%)	4 (28.6%)	0 (0.0%)	4 (19.0%)	0.052^e^
Is the order providing job opportunities for pharmacists?	0 (0.0%)	0 (0.0%)	0 (0.0%)	0 (0.0%)	0 (0.0%)	0 (0.0%)	0 (0.0%)	–^e^
Are all sectors getting the needed attention?	11 (9.6%)	7 (14.0%)	1 (9.1%)	3 (18.8%)	0 (0.0%)	1 (8.3%)	0 (0.0%)	0.221^e^
Does the Sector Need More Regulations and Orientation from the OPL?	111 (97.4%)	48 (96.0%)	10 (90.9%)	16 (100.0%)	14 (100.0%)	12 (100.0%)	21 (100.0%)	0.592^e^
Does the Sector Need More Governmental Support?	106 (93.0%)	45 (90.0%)	10 (90.9%)	14 (87.5%)	14 (100.0%)	12 (100.0%)	21 (100.0%)	0.392^e^
Does the Sector Need More Acknowledgment from Other Healthcare Professionals?	111 (97.4%)	48 (96.0%)	10 (90.9%)	16 (100.0%)	14 (100.0%)	12 (100.0%)	21 (100.0%)	0.594

^a^Hospital pharmacists include clinical pharmacists and employees of the hospital's pharmacy;

^b^Industrial pharmacists include pharmacists working in production, quality assurance and control, research and development, and scientific offices;

^c^Other sectors include academic pharmacists, governmental positions, NGOs positions;

^d^Not in healthcare are either unemployed or are working in non-healthcare institutions;

^e^Chi-square test

^*^Numbers in bold are statistically significant results

Most pharmacists considered that universities and the OPL were poorly involved in providing good professional orientation and that more attention and support are needed from the OPL, the government, and other healthcare professionals.

Additional results varied according to the field of practice as follows: internships were considered sufficient by a higher percentage of hospital pharmacists versus those from other fields, particularly industrial pharmacists (*p* = 0.008); industrial pharmacists and medical representatives felt their positions threatened by non-pharmacists more often than their colleagues in other fields (*p* = 0.001).

Hospital pharmacists and medical representatives considered more often that they had an appropriate orientation in their universities (*p* = 0.052). All the interviewed pharmacists reported that the OPL did not provide jobs for pharmacists and considered that the OPL and other authorities needed to work more efficiently on policies and regulations.

### Professional satisfaction

The main domains assessed for pharmacy job satisfaction were the working environment, financial situation, incentives, and recognition. Overall, almost half of the respondents (48.2%) were poorly satisfied with their job.

The results showed that half of the participants were not psychologically satisfied with their jobs. The various reasons included work relationships, underappreciation of contributions, and talent misuse, the latter being the main reason for dissatisfaction (64.0%). However, psychological satisfaction varied according to the work sector of pharmacists, where a positive correlation was seen in the industrial sector. Industrial pharmacists were the most heard and valued versus other pharmacists, and their competencies were fully utilized (*p* < 0.05).

Most participants were financially dissatisfied. Among community pharmacists, 62.0% were willing to accept jobs unrelated to their specialty to achieve better financial stability. Industrial pharmacists received relatively higher wages (71.4%) compared to those from other fields, especially community pharmacists, who were the least satisfied financially (16.0%; *p* < 0.001). Medical representatives reported not being easily promoted compared to others (*p* = 0.002).

Most pharmacists working in hospitals (72.2%) and industries (71.4%) were more satisfied with their work environment (*p* = 0.03). They also confirmed that tasks were shared between enough employees, making the workload softer and bearable (*p* = 0.006). However, participants working in community pharmacies, medical promotion, and other than the previously mentioned sectors reported being less satisfied with this regard (Table 3).

**Table 3:** Affirmative opinions regarding professional satisfaction

Variable	Total pharmacists	Community pharmacists	Hospital pharmacists^a^	Medical representatives	Industrial pharmacists^b^	Other sectors^c^	Not in healthcare^d^	*p*-value
Psychological satisfaction								
*N* (%)	114 (100.0%)	50 (40.3%)	11 (8.9%)	16 (12.9%)	14 (11.3%)	12 (9.7%)	21 (16.9%)	
Are you satisfied with the on-the-job relationships?	58 (50.9%)	26 (52.0%)	5 (45.5%)	9 (56.3%)	11 (78.6%)	5 (41.7%)	7 (33.3%)	0.182^e^
Are you heard and valued for your contributions?	62 (54.4%)	28 (56.0%)	6 (54.5%)	10 (62.5%)	12 (85.7%)	5 (41.7%)	5 (23.8%)	**0.008**^**e**^
Are your talents fully utilized?	41 (36.0%)	20 (40.0%)	2 (18.2%)	5 (31.3%)	10 (71.4%)	3 (25.0%)	3 (14.3%)	**0.009**^**e**^
Do you consider yourself doing the professional job you enjoy?	58 (50.9%)	23 (46.0%)	7 (63.6%)	7 (43.8%)	12 (85.7%)	6 (50.0%)	8 (38.1%)	**0.07**^**e**^
Financial satisfaction								
Are you willing to work outside your specialty if financially unsatisfied?	66 (57.9%)	31 (62.0%)	5 (45.5%)	7 (43.8%)	8 (57.1%)	8 (66.7%)	13 (61.9%)	0.74^e^
Can you be easily promoted in your current job?	43 (37.7%)	21 (42.0%)	5 (45.5%)	4 (25.0%)	11 (78.6%)	3 (25.0%)	3 (14.3%)	**0.002**^**e**^
Is your salary appropriate?	33 (28.9%)	8 (16.0%)	4 (36.4%)	9 (56.3%)	10 (71.4%)	2 (16.7%)	5 (23.8%)	**< 0.001**^**e**^
Work environment satisfaction								
Is there a suitable working environment?	53 (46.5%)	23 (46.0%)	8 (72.7%)	8 (50.0%)	10 (71.4%)	4 (33.3%)	5 (23.8%)	**0.030**^**e**^
Are there enough employees to cover the workload?	51 (44.7%)	21 (42.0%)	7 (63.6%)	6 (37.5%)	12 (85.7%)	3 (25.0%)	6 (28.6%)	**0.006**^**e**^

^a^Hospital pharmacists include clinical pharmacists and employees of the hospital's pharmacy

^b^Industrial pharmacists include pharmacists working in production, quality assurance and control, research and development, and scientific offices

^c^Other sectors include academic pharmacists, governmental positions, NGOs positions

^d^Not in healthcare are either unemployed or are working in non-healthcare institutions

^e^Chi-square test

^*^Numbers in bold are statistically significant results

### Economic crisis and employment status

Of the total sample, 65.79% of participants felt that the economic crisis and the consequent medication shortages affected their credibility and how society perceived them. All pharmacists were disappointed with the actions of the OPL and the Ministry of Public Health (MOPH) and their lack of support during the current collapse of the sector. Also, 50.88% are planning to leave the country.

About half of the participants (45.6%) reported that their employment status was not affected by the crisis; the rest mainly got a second job to cover their expenses (15.8%) or changed jobs (14.9%). Five of the 18 respondents who took on extra work had a second job unrelated to their profession. Additionally, six pharmacists worked outside the healthcare sector (Table 4).

**Table 4:** The effects of the economic crisis on work status among Lebanese pharmacists

Economic crisis effect on work status	I couldn’t find a job in the healthcare	I lost my job	I had to work a second job to cover my expenses	I changed jobs	My employment status wasn’t affected	Others^a^	*p* value^b^	Total
*N* (%)	3 (2.6%)	6 (5.3%)	18 (15.8%)	17 (14.9%)	52 (45.6%)	18 (15.8%)		114 (100.0%)
Work status							**< 0.001**	
Unemployed	3 (37.5%)	3 (37.5%)	0 (0.0%)	0 (0.0%)	0 (0.0%)	2 (25.0%)		8 (7.0%)
Active	0 (0.0%)	2 (2.1%)	18 (18.6%)	17 (17.5%)	52 (53.6%)	8 (8.2%)		97 (85.1%)
Working abroad	0 (0.0%)	1 (11.1%)	0 (0.0%)	0 (0.0%)	0 (0.0%)	8 (88.9%)		9 (7.9%)

^a^Others include: having to leave the country, struggling to keep running the pharmacy, extra work but lower income

^b^Chi-square

The unemployment rate across our population was low (7.02%). Among currently unemployed pharmacists, 37.5% could not find a job in the healthcare sector, 37.5% lost their jobs because of the economic crisis, and 25.0% left the country.

## Discussion

Our findings revealed that most Lebanese new graduate pharmacists are working in community pharmacies, including those holding a postgraduate degree, given the limited opportunities in other sectors and the unremittingly increasing number of fresh graduates without any control. These results are similar to those of a previous study from Lebanon, showing that poor pharmacy workforce planning and regulation were significantly weakening the profession in Lebanon and that the demand for pharmacists is focused on the community pharmacy sector at the expense of other sectors [18].

The high ratio of pharmacists declaring they are not working in their preferred fields indicates the lack of strategy and governance in pharmacy education. Most respondents felt that the OPL and universities should be more involved in orienting students and providing job opportunities [19] to decrease unemployment. Pharmacists also believed that the OPL and the government should set a limit to prevent non-pharmacists from filling pharmacist positions, especially in community pharmacy and medical promotion, thereby increasing job opportunities for pharmacists. Our results emphasize the need for a national strategy related to education and the workforce, matching the competencies of graduates to the market demands while considering the Lebanese context.

Satisfaction levels varied in our sample, similar to a previous study [12]; the highest was reported among industrial pharmacists, while community pharmacists recorded the lowest. Work environment-related satisfaction was low among community pharmacists since they are subjected to heavy workloads and responsibilities and understaffing, which is expected to negatively affect patient services in the community. Indeed, the dissatisfaction of community pharmacists with their work has shown to be related to incorrectly delivered medications, negligence in detecting adverse drug reactions, and poor patient contact [20], while job satisfaction is an indicator of professionalism, crucial for providing safe and effective care [14]. Nevertheless, most pharmacists working in hospitals, industries, and medical promotion were satisfied with their job environment, given their higher wages, fewer working days, and their right to get holidays. These positions seem mainly for pharmacists with an older date of graduation.

Our findings also showed that pharmacists were not satisfied with their financial situation, particularly those working in community pharmacies, likely due to decreased sales, profits, number of loyal customers, the lack of career advancement, and non-pharmacists filling their position with lower wages, particularly during the current crisis. Undoubtedly, a higher income improves personal satisfaction with work, explaining why pharmacists were more often subjected to job turnover [14]. Overall, these results may indirectly imply that fresh graduates are less satisfied with their positions in the community than older pharmacists in other sectors.

Furthermore, the crisis facing the country has not only resulted in some pharmacists losing their jobs but also caused some to seek a life abroad and others to work double shifts. This unparalleled economic decline has led pharmaceutical companies to reduce their workforce [21]. In contrast, some pharmacists were not affected by the crisis since the COVID-19 pandemic made people rush into pharmacies, thus increasing their workload [22, 23].

As for the suggestions for authorities, it is paramount that the OPL develops and executes a national workforce strategy, in collaboration with the MOPH and in accordance with the FIP strategy, to improve the pharmacy profession since its stated mission is to protect the rights of pharmacists and maintain the excellence of the pharmacy profession through implementing rules and regulations and promoting scientific competencies [24]. Upscaling the pharmacy profession requires dedicated and coordinated efforts from all interested parties, which would improve the professional image of pharmacists and allow for better pharmaceutical services in Lebanon.

The OPL should also suggest new laws to concerned authorities and apply existing ones, such as the one related to the clinical pharmacy in hospital settings. It should require the presence of clinical pharmacists in each department and every floor round and specify the number of pharmacists needed per hospital bed [25]. The law penalizing companies and institutions employing non-pharmacists in positions entitled to pharmacists should be enforced. Moreover, the OPL should specify a minimum monthly wage calculated per working hours, susceptible to change according to the economic and social status of the country [26]. It should also ensure the application of these laws by conducting regular inspection rounds to pharmaceutical establishments and applying penalties in case of noncompliance. Moreover, despite comprehensive, evidence-based strategic plans, including surveys and epidemiological research, previously developed and endorsed by the OPL, none has yet been implemented by health authorities, such as the MOPH [27]. Existing projects should be implemented in the short term; policies and regulations regarding pharmacy curricula reforms, accreditation, post-graduate training, recognition of pharmacy specialties, organizing the profession, and assessing advanced competencies should all be updated and applied [28].

Moreover, it is essential to give more attention to undergraduates and new graduates to reduce the mismatch between education and practice fields while guaranteeing that competencies will be used adequately and optimizing professional performance in every sector [29]. It is also recommended to design continuing education programs that address local needs, such as soft skills in management and leadership for community pharmacists or marketing and interpersonal skills for medical representatives, for example [30]. Hence, developing a national competency framework, in collaboration with the Ministry of Education and Higher Education, to be implemented in pharmacy schools is essential to guide pharmacy education [31].

Furthermore, the number of pharmacy graduates should be decreased by enforcing maximum acceptance quotas in pharmacy schools and making the national competency assessment examination more challenging [19]. Additionally, since healthcare professionals are expected to operate in multidisciplinary teams, favoring interprofessional collaboration within and across settings would provide the best results for patients while minimizing errors and costs [32]. Reforms at the professional and educational levels are supported by the fact that the area of expertise and jobs of health practitioners have changed and need to conform to modern care standards [32].

Moreover, research related to pharmacy education matters is recommended, since it is lacking in the developing countries (17% of global research) [33]; in addition, improving the pharmacy practice conditions is of primary necessity, with many conditions specific to developing countries that may not be similar to those of developed countries [34].

### Limitations

This pilot study has several limitations. First, the sample was relatively small and cannot be representative of the entire Lebanese pharmacist population; future studies taking into account the distribution of pharmacists on different sectors is suggested. Second, a significant number of pharmacists might have eluded filling out the questionnaire once asked about OPL registration since a proportion of pharmacists are not declaring their active working status because of registration and retirement fees. Third, older pharmacists could have found it annoying to complete an online survey and merely ignored it. Furthermore, because the data were acquired via a self-administered questionnaire, the results might be biased, as a non-objective understanding of questions is possible, especially those related to career perception and job satisfaction. Further large-scale studies using validated tools are necessary for an in-depth assessment of the pharmaceutical workforce distribution and current status in Lebanon; future projections also need to be conducted.

## Conclusion

Our study findings revealed that most Lebanese fresh graduates worked as community pharmacists, which does not correspond to their preference, the available job market, and the modern pharmacy wingspan. Most pharmacists were financially, physically, and emotionally dissatisfied due to the Lebanese economic crisis that added heavy workloads and responsibilities without any pecuniary raise. Unified efforts are needed from all stakeholders to improve workforce planning, optimize the integration of pharmacists into work sectors that need it, and improve the financial, physical, and emotional well-being of pharmacists in Lebanon.

## Data Availability

The datasets used and/or analyzed during the current study are available from the corresponding author on reasonable request.

## Abbreviations

WHO: World Health Organization
FIP: International Pharmaceutical Federation
OPL: Order of Pharmacists of Lebanon
SPSS: Statistical Package for the Social Sciences
MOPH: Ministry of Public Health
COVID-19: Coronavirus disease 2019
